# Disparities along the glioblastoma clinical trials landscape

**DOI:** 10.1093/neuonc/noy176

**Published:** 2018-11-26

**Authors:** Ethan B Ludmir, Jacob J Mandel, Mary Frances McAleer, John F de Groot

**Affiliations:** 1 The University of Texas MD Anderson Cancer Center, Houston, Texas; 2 Baylor College of Medicine, Houston, Texas

We read with interest the recent work by Vanderbeek et al^1^ regarding the current clinical trials landscape for glioblastoma (GBM) patients. An unexplored dimension of their analysis centers on disparities and demographic discrepancies between clinical trial participants and the broader GBM population. We therefore examined clinical trials with published results as highlighted by the authors, totaling 51 trials.^1^ While most of these trials reported details regarding patient age (48/51, 94%) and gender (47/51, 92%), only 14 trials (27%) provided information regarding ethnicity and/or race in either peer-reviewed publications or ClinicalTrials.gov. The rate of reporting ethnicity/race was particularly low among phase I/II studies (9/43, 21%) compared with phase III trials (5/8, 63%, chi-squared test *P* = 0.02).

The demographic composition of GBM trial participants poorly reflects the broader GBM population in the United States. For trials reporting results, average median age at diagnosis of trial participants was 55.3 years (standard error [SE] 0.63 y); this is significantly younger than the recently published population median age at diagnosis of 63 years (*t*-test, *P* < 0.001).^2^ Similarly, fewer women were enrolled on trials (2414/6292 patients, 38.5%) compared with the overall proportion of female GBM patients (42.7%, *P* < 0.001).^2^ Among those trials providing information regarding ethnicity/race, the proportion of non-Hispanic white (NHW) patients enrolled on protocols was higher than the population average for US GBM patients (population average 86.0%)^2^; this was true whether all trials were included (90.4% NHW patients, *P* < 0.001) or the analysis was limited to trials exclusively with enrollment in the US (90.8% NHW patients, *P* = 0.01).

To assess if any specific subgroup of trials were at increased risk for demographic skew, the roles of industry funding and trial phase were analyzed. Twenty-six trials (51%) reported pharmaceutical industry funding; these industry-funded trials had a smaller proportion of female patients than non-industry funded trials (37.4% vs 40.3%, *P* = 0.02). Furthermore, for trials accruing in the US only, industry-funded trials included a higher proportion of NHW patients compared with non-industry trials, though this difference was not statistically significant (93.9% vs 89.5%, *P* = 0.21). As previously noted, phase III trials reported ethnicity/race at a significantly higher rate than phase I/II trials (63% vs 21%, *P* = 0.02). As no phase III trials enrolled exclusively in the US, we compared the racial demographics of the study populations for those trials accruing a majority of patients from the US; comparing these trials, we found that phase III trials enrolled a higher rate of NHW patients than phase I/II studies (94.2% vs 91.2%, *P* = 0.02). These findings suggest higher rates of demographic bias among both industry-funded studies as well as phase III trials.

We commend the authors for their article assessing the current GBM trial portfolio; building upon these efforts, the above data represent the most robust evidence to date reporting on disparities among GBM trial participants.^3^ GBM patients who enroll on clinical protocols are more likely to be younger, male, and NHW, echoing demographic trends observed among trial patients with other malignancies such as colorectal and lung cancer.^4^ As these demographic factors have been associated with differential survival and outcomes,^2,5^ advocating for representative trial samples is critical to supporting generalizability of trial results. Given the dismal prognosis of GBM and the poor conversion rate of positive early-phase trials into positive phase III trials,^6^ we encourage authors to more consistently report trial demographics, particularly as regards ethnicity/race. This need to provide demographic information is especially true in the context of GBM, where NHW patients have a worse prognosis than minority groups.^2,5^ Such efforts can better facilitate identification of subgroups that may benefit from novel interventions while improving trial access equity for all GBM patients.

## Funding

There are no funding sources for this work.

**Conflict of interest statement.** None declared.
